# Alteration of Blood Flow in a Venular Network by Infusion of Dextran 500: Evaluation with a Laser Speckle Contrast Imaging System

**DOI:** 10.1371/journal.pone.0140038

**Published:** 2015-10-14

**Authors:** Bumseok Namgung, Yan Cheng Ng, Jeonghun Nam, Hwa Liang Leo, Sangho Kim

**Affiliations:** 1 Department of Biomedical Engineering, National University of Singapore, Singapore; 2 NUS Graduate School for Integrative Sciences and Engineering, National University of Singapore, Singapore; 3 Department of Surgery, National University of Singapore, Singapore; Université Claude Bernard Lyon 1, FRANCE

## Abstract

This study examined the effect of dextran-induced RBC aggregation on the venular flow in microvasculature. We utilized the laser speckle contrast imaging (LSCI) as a wide-field imaging technique to visualize the flow distribution in venules influenced by abnormally elevated levels of RBC aggregation at a network-scale level, which was unprecedented in previous studies. RBC aggregation in rats was induced by infusing Dextran 500. To elucidate the impact of RBC aggregation on microvascular perfusion, blood flow in the venular network of a rat cremaster muscle was analyzed with a stepwise reduction of the arterial pressure (100 → 30 mmHg). The LSCI analysis revealed a substantial decrease in the functional vascular density after the infusion of dextran. The relative decrease in flow velocity after dextran infusion was notably pronounced at low arterial pressures. Whole blood viscosity measurements implied that the reduction in venular flow with dextran infusion could be due to the elevation of medium viscosity in high shear conditions (> 45 s^-1^). In contrast, further augmentation to the flow reduction at low arterial pressures could be attributed to the formation of RBC aggregates (< 45 s^-1^). This study confirmed that RBC aggregation could play a dominant role in modulating microvascular perfusion, particularly in the venular networks.

## Introduction

Microvasculature provides a large area that allows the exchange of substances between the blood stream and surrounding tissues. Thus, microcirculation plays a fundamental role in regulating tissue oxygenation, nutrients supply and eventually organ survival. As blood continuously circulates the entire human body while virtually reaching to all peripheral regions, tissue perfusion becomes an important indicator for evaluating the hemodynamic microvascular function. Accordingly, impaired tissue perfusion can potentially result from abnormal alteration in the rheological properties including red blood cell (RBC) aggregation, deformability and hematocrit. Among these factors, RBC aggregation has gathered great attention [1–5] due to its clinical relevance to various disease conditions [6–9]. Thus, it is important to understand the relation between RBC aggregation and flow resistance in the microvascular network since the latter is inversely proportional to the perfusion.

In many previous *in vivo* and *in vitro* studies, Dextran 500 has been used to induce RBC aggregation. Previous *in vitro* studies performed in a vertical glass tube reported that the formation of near-wall cell-free layer resulted in a reduction in the apparent viscosity [10,11]. In contrast, flow resistance could increase with RBC aggregation at low shear rates in a horizontal tube due mainly to sedimentation of RBCs [12]. Unlike the *in vitro* micro-tube experiments, the microvascular network has a complex geometry with multiple branches. Therefore, the effect of RBC aggregation on vascular resistance may not be adequately examined from the findings obtained in *in vitro* studies [13,14]. Moreover, a previous *in vivo* study highlighted that the tissue perfusion could decrease with RBC aggregation [3]. The increase in flow resistance due to RBC aggregation is prominent in venules due to their low shear condition which is favorable for the formation of RBC aggregates. Tissue perfusion was found to be significantly modulated at reduced arterial pressures (50 and 25 mmHg) with RBC aggregation when evaluated by the functional capillary density (FCD) [3]. However, most previous studies relied only on data obtained from a single-vessel or capillary-based analysis, thus greatly limiting the ability to highlight the effect of RBC aggregation on microvascular network flows.

Recently, *in vivo* studies have adopted the laser speckle contrast imaging (LSCI) technique to visualize the microvascular remodeling and hemodynamic changes [15,16]. LSCI provides high-resolution perfusion images in a relatively wide field by analyzing laser speckle patterns with time. Owing to the great advantages of LSCI including low-cost, ease of setup, and noninvasive measurement, it has been widely used in various studies on cerebral blood flow [17,18], retinal blood flow [19,20], liver microcirculation [21], and wound healing angiogenesis [15]. In addition, a recent study proposed the use of speckle contrast imaging to quantify the functional vascular density (FVD) [22] which has been used to examine changes in blood flow. In the present study, we adopted the LSCI to visualize the microvasculature of the rat cremaster muscle to obtain quantitative flow information at a network scale. Particularly, this study aimed to elucidate how the blood flow in venules at the network level is altered by the arterial pressure reduction and/or the aggregation induced by Dextran 500.

## Materials and Methods

### Animal preparation

All animal handling procedures in this study have been approved by the *National University of Singapore Institutional Animal Care and Use Committee* (approved protocol no. 098/12). A total of ten male rats (Sprague-Dawley) weighing 197 ± 19 g were used in this study. The animals were initially anesthetized with ketamine (37.5 mg/ml) and xylazine (5 mg/ml) cocktail through intraperitoneal injection (2 ml/kg). The surgery was performed on a heating pad to maintain the animal body temperature at 37°C. The animal was tracheotomized and the jugular vein was catheterized for the administration of additional anesthetic and dextran solutions during the experiment. The femoral artery was catheterized for blood sample withdrawal and real-time pressure monitoring. All catheters were heparinized with saline (30 IU/mL) solution to prevent blood clotting. The rat cremaster muscle was surgically exposed, and stretched on a customized animal platform with two heating elements to maintain the muscle temperature at 35°C. At the end of experiments, the animal was euthanized with an overdose of pentobarbital sodium.

### Induction of RBC aggregation

Dextran 500 (Avg. MW = 450–550 kDa, Pharmacosmos A/S, Denmark) was first dissolved in saline (60 mg/ml). A total of 250 mg/kg was infused into rats over the course of 1–2 min through the jugular vein to achieve a plasma-dextran concentration of ~0.78% in the rat blood [4]. There was no discernable adverse reaction (e.g., visible swelling of the limbs) to the Dextran 500 infusion in the rats used in this study.

### Hematocrit and viscosity measurement

A blood sample (~0.1 ml) was withdrawn from the femoral artery before and 15-min after the dextran infusion for the hematocrit measurement (Sigma 1–14 Microcentrifuge, Sigma, Germany). The whole blood viscosity was measured at 35°C over a range of shear rates ($\dot{\gamma }$ = 11.25 − 90 s^-1^) before and after the dextran infusion with a cone-and-plate viscometer (DV-II+, Brookfield Engineering Laboratories, Inc., Middleboro, MA, USA). Similarly, blood plasma viscosity was measured after extraction, from a blood sample by centrifugation (Sigma 2–6, Goettingen, Germany).

### Laser speckle contrast imaging

**Fig 1:**
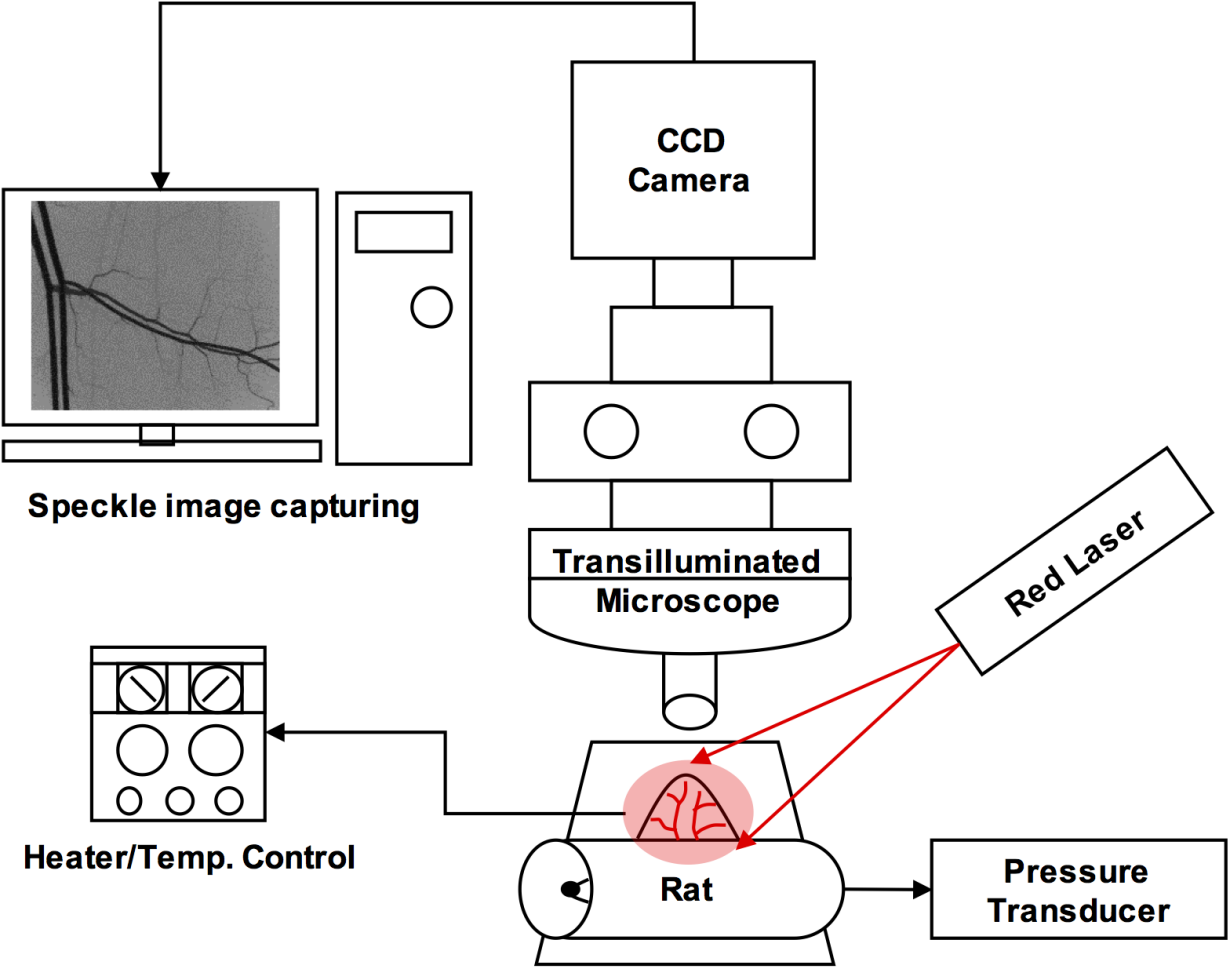
Schematic diagram of intravital microscopic laser speckle contrast imaging system (LSCI).

Fig 1 shows the schematic of our intravital microscopic laser speckle contrast imaging (LSCI) system used to visualize the microvascular flow of the rat cremaster muscle. The rat was placed on the microscopic stage and allowed to stabilize over a period of ~10 min. The arterial cannulation was connected to a physiological data-acquisition system (MP 100 System with TSD 104A, BIOPAC Systems, USA) for continuous pressure monitoring during the experiments. Raw speckle images of microcirculation were acquired with an adjustable focus laser module (CPS196, 635 nm, 4.5mW, Thorlabs Inc., New Jersey, USA) and a high-sensitive sCMOS camera (Pco.edge, PCO AG, Kelheim, Germany) mounted on an intravital microscope (BX51, Olympus, Japan) with a 4X objective (UPlanFl, NA = 0.13, Olympus, Japan). The laser diode was equipped with a micromanipulator (NMN-21, Narishige, Japan) on the fixed microscopic stage platform for constant illumination.

The microscopic system is capable of providing images at 1280 x 1080 pixels with a pixel resolution (*ρ*_pixel_) of 3.22 μm/pixel. To satisfy the Nyquist criterion [23], the speckle size (*ρ*_speckle_) was ensured to be at least two times greater than the image pixel size (*ρ*_speckle_ ≥ 2*ρ*_pixel_). The speckle size can be given by *ρ*_speckle_ = 2.44*λ*(1+M)*f*/#, where *λ* is the wavelength of the laser light, M is the magnification of the imaging system, and *f*/# is the *f* number of the system [24]. The speckle size relative to the image pixel size in the present study was ~9 pixels per speckle. The light intensity was carefully adjusted to match the dynamic range of the camera during the image recording for consistent image quality. The speckle contrast images were recorded at 10 frame/s to obtain a total of 80 images with a fixed exposure time of 10 ms.

**Fig 2:**
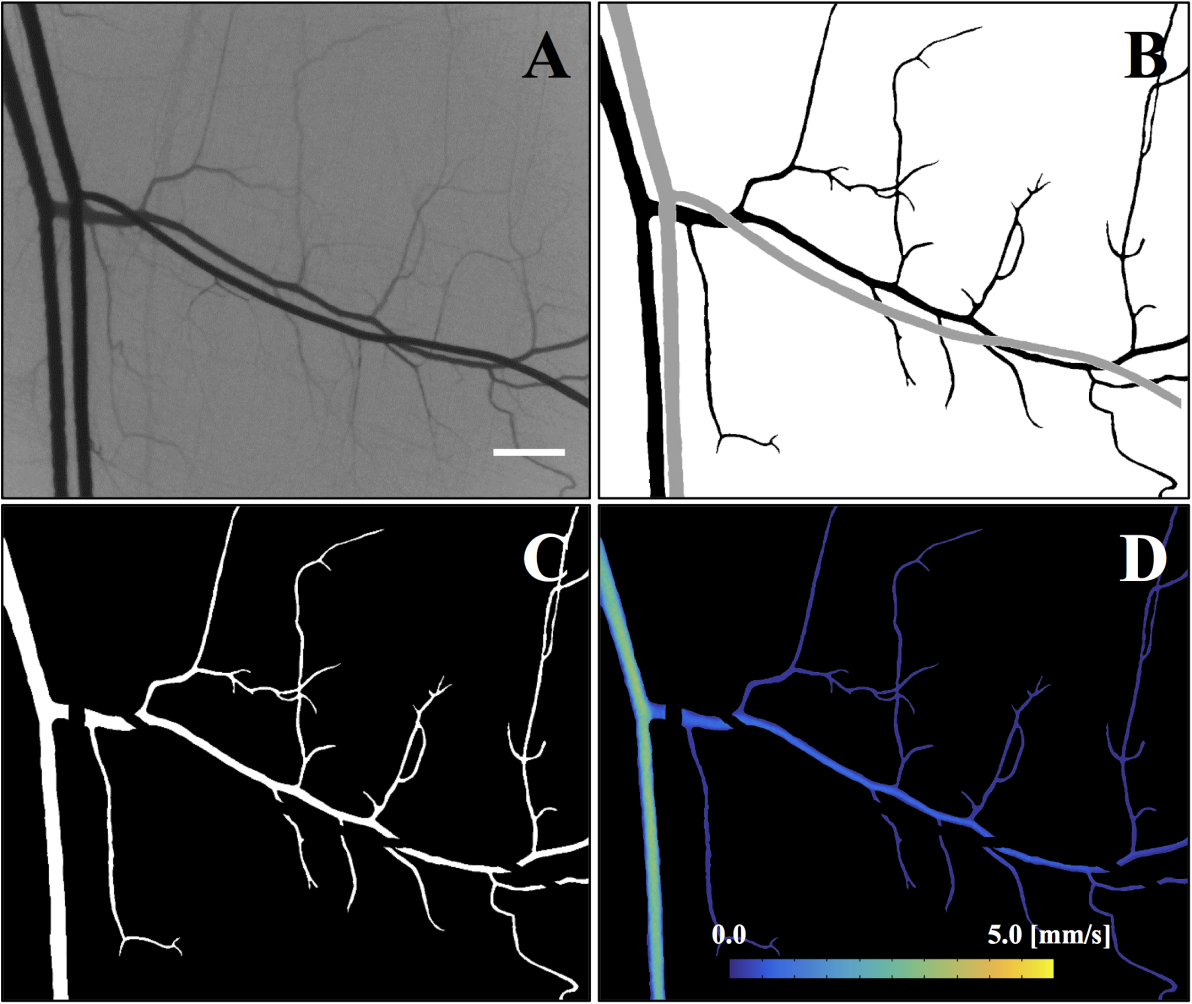
Velocity map of venular network flow. **A:** Speckle contrast image obtained at normal arterial pressure (MAP = 100 mmHg). **B:** Binary image for the vessel mask (Black: venules, Gray: arterioles). **C:** Venular vessel mask. **D:** Venular velocity map. Scale bar = 500 μm.

Fig 2 shows a typical example of the microvasculature in the rat cremaster muscle visualized by LSCI. The speckle contrast (*K*) at each individual pixel (*i*, *j*) of the image was obtained by adopting the previously described temporal scheme [25] as follows:

$$K\left(i,j\right)=\frac{\sigma_{80}\left(i,j\right)}{\mu_{80}\left(i,j\right)}$$ (1)

where *μ*_80_ and *σ*_80_ represent the pixel-wise mean and standard deviation over 80 sequential images, respectively. The velocity (*v*) is inversely related to the speckle correlation time (*τ*_c_) [26] which is related to *K* by:

$$K^{2}=\frac{\tau_{c}}{2T}$$ (2)

where *T* represents the exposure time of the camera. This simplified relation was adopted to enhance the image processing performance [20,27]. All images utilized in this study were ensured to satisfy *K* < 0.6 in order for Eq 2 to be valid [27,28] (S1 Fig). All the image processing was performed with a custom-built image processing script using MATLAB (Mathworks, MA).

### Experimental protocol and vessel mask preparation

The mean arterial pressure was varied from 100 to 30 mmHg by partially occluding the abdominal aorta via a surgically inserted pneumatic cuff. The aortic occlusion was repeated for each pressure condition and maintained for 1 min before the recording of images. A vascular region, where the venular vessel branching was substantial and clearly focused, was selected for the recording. The field of view was then fixed during the course of the experiment after confirming the region of interest.

The functional vessel region was generally less than 10% of the entire field of view in the present study, thus an average value for the velocities over the entire field of view might not well represent the flow in the microvasculature. Hence, a vessel mask technique was utilized to isolate the venular network of interest for this study (Fig 2). A semi-automated image processing was performed on the speckle contrast image (*K*) acquired at the normal mean arterial pressure (MAP ~100 mmHg, Fig 2A) to obtain a representative venular network mask (Fig 2C). Accordingly, all the vessels were assumed to be functional at the normal MAP. The contrast image was subsequently converted into a binary image (Fig 2B) by utilizing an adaptive thresholding algorithm [29]. Small isolated objectives (number of pixels < 100) were considered as noise and removed from the binary image. Additionally, discontinuities in the vessels were manually compensated by collating with the original speckle contrast image in Fig 2A. Arteriolar vessels were then identified and removed from the venular network mask (Fig 2B and 2C). Concomitantly, the physical velocities were estimated by calibrating our LSCI measurements with *in vitro* experiments using predetermined flows in microfluidic channels. The details of the calibration experiments can be found in S1 Text. Finally, the venular network mask was overlaid onto the original velocity map to acquire the velocity map for the venular network (Fig 2D). As, also reported previously [30], there was no significant change in the diameter of the venules with a reduction in the MAP. Hence, the representative venular vessel mask obtained at the normal MAP (Fig 2C) was then applied to images obtained in all other pressure conditions to acquire the respective velocity maps.

### Functional vascular density

To evaluate the impairment of vascular perfusion by a reduction in MAP and/or infusion of Dextran 500, we employed the LSCI to quantify the functional vascular density (FVD) in the rat muscle as reported in an earlier study [22]. The FVD is an indicator of identifying the functionality of vessels with blood flow since the flow can be visualized by LSCI based on the movement of RBCs [22]. The speckle contrast image was smoothened using the Gaussian filter and converted into a binary image (Figure B in S3 Fig). The binary image was then skeletonized until a single pixel remained across the vessel diameter, and three pixels at each branching point were removed to segment each vessel (Figure C in S3 Fig). Consequently, the FVD was calculated by dividing the total pixels over all vessel segments (total length of vessels) with the total pixels in the field of view. It is important to note that previous studies reported no observable change in the venular diameter during a stepwise arterial pressure reduction to 20 mmHg in the cat sartorius skeletal muscle [31] and with arterial pressure reduction to 40 mmHg in the rat spinotrapezius muscle [30]. In addition, no change in the venular diameter was observed with Dextran 500 infusion at a plasma-dextran concentration of ~0.6%. [30]. These findings are in consensus with ours when using a dextran concentration of ~0.78% (S4 Fig). There was no significant change in the venular diameter due to the dextran infusion or flow reduction.

### Statistical analysis

A statistical software package (Prism 6.0, GraphPad) was used for all statistical analyses. Paired *t*-test with a two-tailed test was performed for comparison of two groups (before and after dextran infusion) for FVD and relative velocity decrease at each MAP, whereas unpaired *t*-test was performed for the viscosity comparison that was made from two separated groups of animals. *P* < 0.05 was considered statistically significant. Linear regression was performed to examine the statistical significance in the relation between two variables. The relation was considered to be statistically significant when the slope of regression line was significantly deviated from zero (*P* < 0.05). In addition, the analysis of covariance (ANCOVA) was performed to evaluate whether the difference between the slopes is significant.

## Results and Discussion

### Systemic conditions

The mean arterial pressures (MAP) and hematocrits are listed in Table 1. There was no statistical difference in the systemic parameters before and after dextran infusion.

**Table 1:** Systemic parameters.

Dextran infusion	Hct [%]	MAP [mmHg]							
Before	43 ± 1	30.6 ± 2.0	40.3 ± 0.4	50.3 ± 0.4	60.4 ± 0.7	70.5 ± 0.5	80.0 ± 0.6	90.2 ± 0.6	101.0 ± 1.7
After	42 ± 1	30.9 ± 1.7	40.1 ± 0.3	49.9 ± 0.7	60.2 ± 0.5	70.1 ± 0.7	79.9 ± 0.5	89.8 ± 0.6	100.5 ± 0.6

### Calibration and validation of velocity measurement

**Fig 3:**
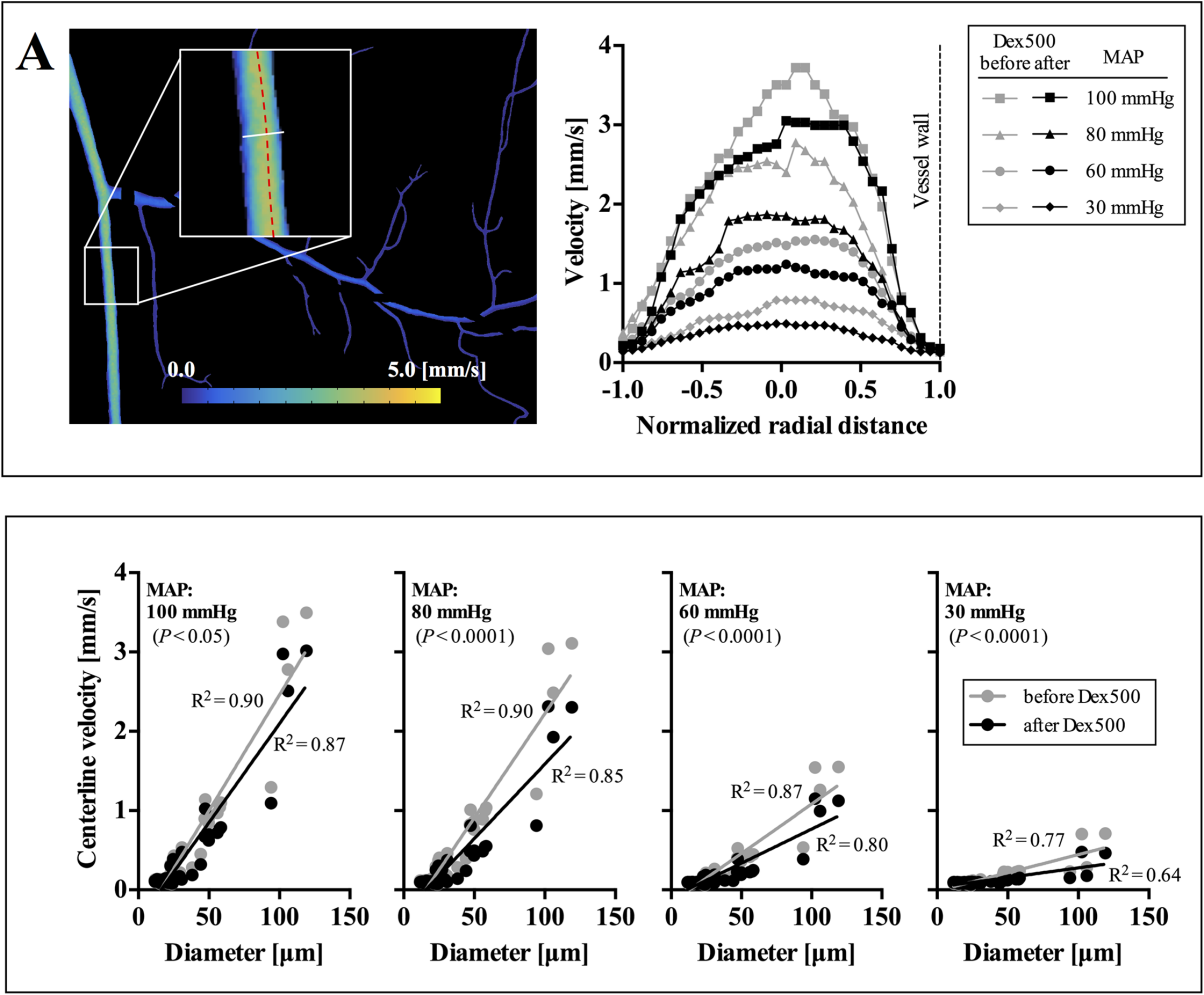
Typical example of velocity profile and distribution in the venular network. **A:** Representative velocity profiles at various MAPs in a venule (ID ~95 μm). **B:** Centerline velocity as a function of vessel diameter at various MAPs (total 55 vessel segments). Data were obtained from the single venular network shown in **A**. Solid lines represent the linear regression fits. *P*-values represent the statistical significance in the difference between the slopes of regression lines before and after dextran infusion.

Typical examples of venular velocity profiles before and after dextran infusion at different MAPs are shown in Fig 3A. The centerline velocities (*V*_c_) determined in this study were in agreement with the range observed in a 50-μm venule by Bishop et al. [32] at 120 mmHg (*V*_c_ = 4.5–5.8 mm/s) and 50 mmHg (*V*_c_ = 0.41–0.43 mm/s). In addition, Durussel et al. [5] reported a positive relation between RBC velocity and vessel diameter for venules up to 150 μm with corresponding velocities of up to 4 mm/s which was also observed in this study (Fig 3B), thus validating the measurements of venular velocity using our LSCI system.

The decrease in the *V*_c_ was apparent after dextran infusion when compared to that before dextran infusion in all pressure conditions (Fig 3A and 3B). Furthermore, the velocity profiles seemed to be more blunted with dextran infusion and decreasing MAP, which could be due mainly to aggregation. A similar trend in the flow velocity following dextran infusion was also reported in a previous study [1] which examined venular flows at a single-vessel level. Interestingly, as shown in Fig 3B, the centerline velocity in the venules appeared to decrease after dextran infusion for the entire range of vessel size in this study (*D* = 24–120 μm). In particular, the maximum centerline velocity in the venular network declined from 3.50 mm/s to 3.02 mm/s (*D* = 120 μm at MAP = 100 mmHg).

### Effect of dextran infusion on functional vascular density

**Fig 4:**
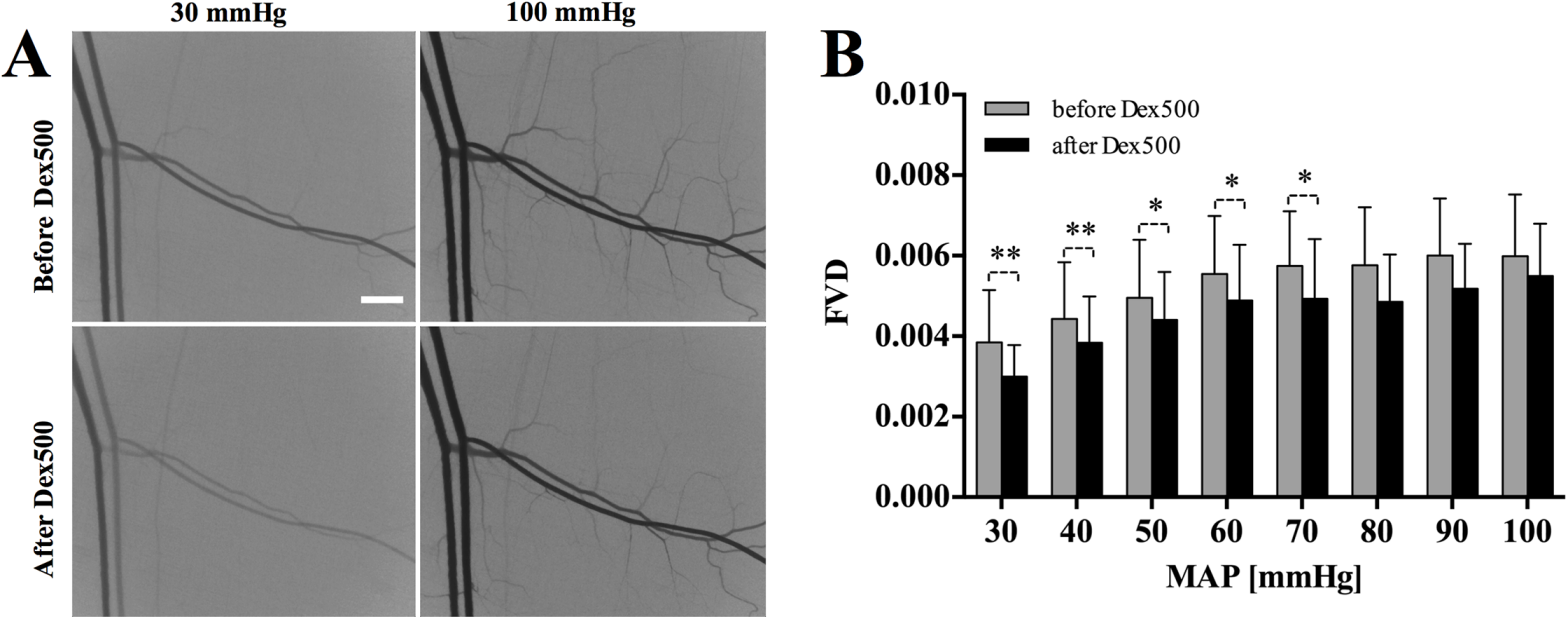
Effect of arterial pressure reduction and dextran infusion on functional vascular density (FVD). **A:** Typical examples of speckle contrast images at 30 and 100 mmHg before and after dextran infusion. **B:** FVD corresponding to the MAP reduction before and after dextran infusion. Scale bar = 500 μm. (* *P* < 0.5, ** *P* < 0.005; significant decrease due to the dextran infusion.)

Fig 4A and 4B show a typical example of the speckle contrast images and the functional vascular density respectively, over a range of MAPs from 30 to 100 mmHg before and after dextran infusion. As illustrated in Fig 4A, the contrast value (*K*) was lower in the reduced MAP condition as compared to that at 100 mmHg. The decline in *K* was due to a decrease in the standard deviation of speckles (*σ*) attributed to the reduction in RBC flow. As expected, there was a positive relation between the functional vascular density (FVD) and MAP regardless of dextran infusion. The FVD after dextran infusion was significantly lower than that before dextran infusion at MAPs below 70 mmHg. Although the difference in FVD before and after dextran infusion was not statistically significant, the mean FVD after dextran infusion was consistently lower than that before dextran infusion over the entire range of MAPs (Fig 4B).

A previous *in vivo* study reported similar findings in the alteration of functional capillary density (FCD) by RBC aggregation [3]. It was reported that even at a lower plasma-dextran concentration (0.6% wt/vol), RBC aggregation could impede the FCD at normal MAP. Moreover, this reduction was more pronounced at a reduced MAP. In the present study, there also seemed to be a trend of reduction in FVD after dextran infusion. In particular, the significant reduction in the FVD after dextran infusion at low MAPs (< 70 mmHg) indicates that RBC aggregation has an important role in reducing RBC flux in the small vessels. It should be noted that although the LSCI system used in this study could not take into account the capillary flow (FCD) due to the limited image resolution (3.22 µm/pixel), we herein demonstrate an overall reduction in microvascular flow by dextran-induced RBC aggregation, particularly in low shear conditions. In contrast, our results suggest that the decrease in the FVD after dextran infusion did not have any apparent relation with the MAP. Since there was no notable difference in reduction of FVD between the low and high shear conditions, RBC aggregation may not have a significant role in influencing the FVD. This could also be partly attributed to the elevated RBC flux in the capillaries resulting from a reduction in the FCD at low MAPs [3]. As such, this also implies that RBC flows in capillaries (and thus the FCD) could be more sensitive to rheological alterations such as aggregation and pressure as compared to the FVD.

### Effect of dextran infusion on venular network flow

**Fig 5:**
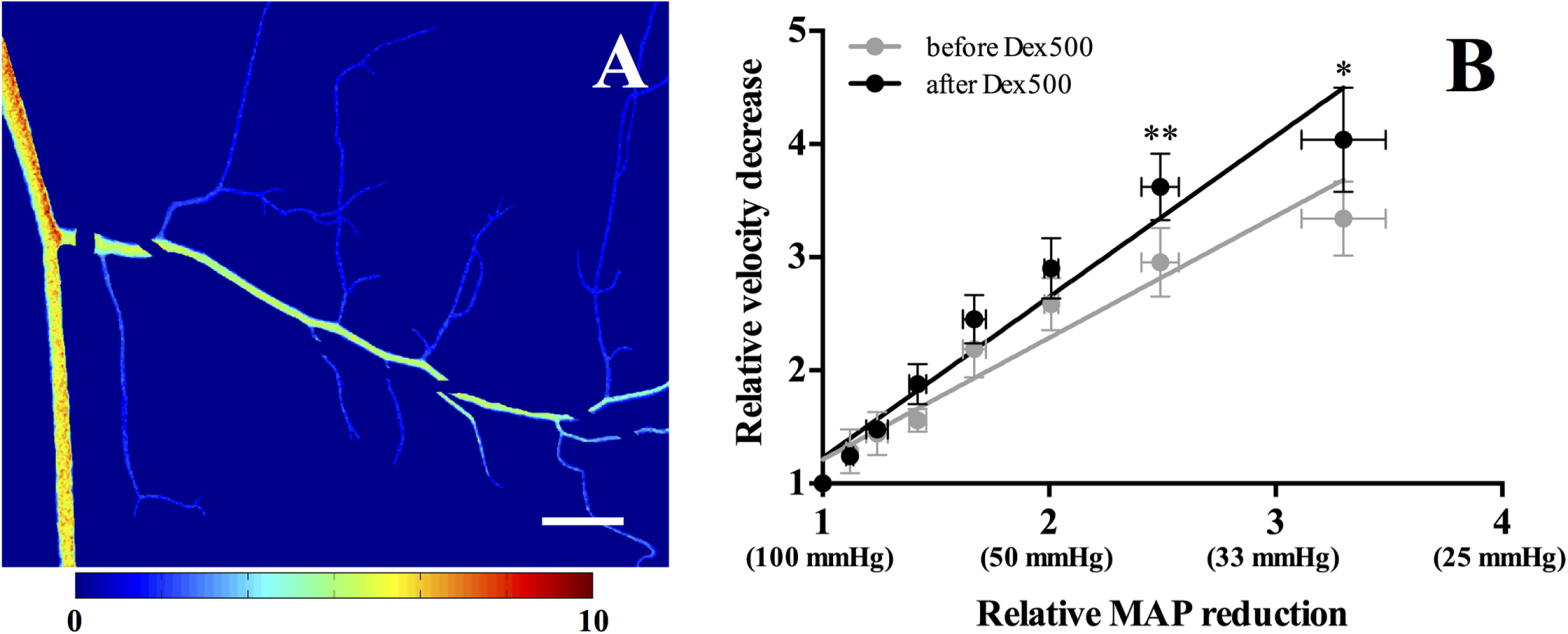
Relative velocity in the venular network. **A:** Typical example of the relative velocity decrease map at 30 mmHg. **B:** Relative velocity decrease at each MAP with respect to the normal arterial pressure (MAP = 100 mmHg). The solid lines represent linear regression fits before (*y* = 1.075*x* + 0.136, *R* ^2^ = 0.58, significance of slope: *P* < 0.0001) and after (*y* = 1.422*x* − 0.196, *R* ^2^ = 0.70, significance of slope: *P* < 0.0001) dextran infusion, respectively. Scale bar = 500 μm.

Fig 5A shows a typical relative velocity map at 30 mmHg which was obtained by performing a pixel-wise comparison with that at the normal MAP (100 mmHg) ($I_{i,j}^{Normal}/I_{i,j}^{P}$, where *P* = 30, 40… 90 mmHg, *i* and *j* = pixel coordinates). In addition, the relative velocities were averaged by the total number of pixels constituting the venules to evaluate the variations of the overall relative velocity in the venular network as shown in Fig 5B. Regardless of dextran infusion, the relative velocity in venules exhibited a significant decreasing trend with declining MAP. The mean relative velocity of the venular network appeared to decrease after dextran infusion. Furthermore, the relative velocity decrease was significantly augmented at low MAPs of 30 (*P* < 0.05) and 40 mmHg (*P* < 0.01). When the MAP decreased to 30 mmHg, the reduction of mean relative velocity after dextran infusion was ~20% greater than that before the infusion. The slopes of regression lines before (*y* = 1.075*x* + 0.136, *R* ^2^ = 0.58) and after (*y* = 1.422*x* − 0.196, *R* ^2^ = 0.70) dextran infusion (Fig 5B) correspondingly exhibited a significant difference (*P* < 0.05).

Previous studies which utilized the cat gastrocnemius muscle showed a similar result in which the venous vascular resistance was found to be inversely related to the blood flow rate [33]. Conversely, this phenomenon was mainly due to the shear-dependent RBC aggregation that is inherent in the cat blood. Another study [1] that had used the rat spinotrapezius muscle with dextran induced RBC aggregation attributed this inverse relation to the enhanced bluntness of the venular velocity profile at low flow rates, which is also highlighted in the present study (Fig 3).

### Effect of dextran infusion on blood viscosity

**Fig 6:**
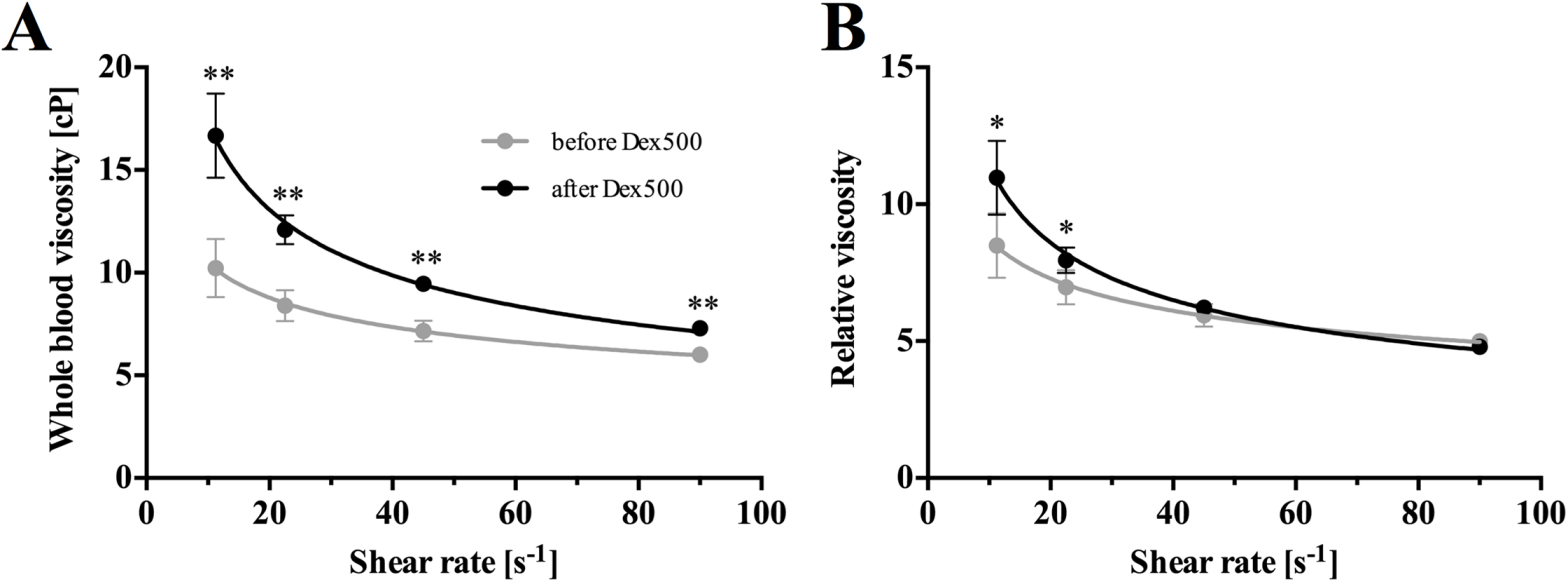
Shear-dependent blood viscosity. **A:** Whole blood viscosity (μ_wb_). **B:** Relative viscosity (μ_rel_) to plasma viscosity (μ_pl_) before and after dextran infusion at 35°C (*n* = 10). The solid lines represent curve fittings by the power-law model ($\mu \;=\;m{\dot{\gamma }}^{n-1}$). (μ_wb, before_: *m* = 18.87, *n* = 0.75, *R* ^2^ = 0.79; μ_wb, after_: *m* = 43.88, *n* = 0.60, *R* ^2^ = 0.91; μ_rel, before_: *m* = 15.67 *n* = 0.75, *R* ^2^ = 0.79; μ_rel, after_: *m* = 28.86, *n* = 0.60, *R* ^2^ = 0.91) (* *P* < 0.001, ** *P* < 0.0001).

The overall increase in whole blood viscosity (μ_wb_) due to the elevated plasma viscosity (μ_pl_) and/or the formation of RBC aggregates after dextran infusion could probably contribute to the reduction in venular flow. Fig 6A shows the shear-thinning behavior of rat blood before (μ_wb, before_) and after (μ_wb, after_) dextran infusion over a range of shear rates ($\dot{\gamma }$ = 11.25 − 90 s^-1^) at 35°C. The curve fitting by the power-law model ($\mu \;=\;m{\dot{\gamma }}^{n-1}$) clearly showed that μ_wb, after_ was consistently greater than μ_wb, before_, and the difference between the two viscosities progressively increased with decreasing $\dot{\gamma }$. To better depict the effect of RBC aggregation on the increase in blood viscosity, the values of μ_wb_ were normalized by μ_pl_ and represented as relative viscosity (μ_rel_) in Fig 6B. μ_rel, after_ was significantly greater (*P* < 0.001) than μ_rel, before_ in low shear conditions (11.25–22.5 s^-1^) whereas no significant difference was observed between them at shear rates greater than 45 s^-1^. Concomitantly, the infusion of dextran increased μ_pl_ from 1.20 ± 0.03 to 1.52 ± 0.01 cP. This implied that the difference in μ_wb_ in high shear conditions ($\dot{\gamma }$ > 45 s^-1^) could be due mainly to the elevation in μ_pl_ with dextran infusion, whereas the elevation in μ_wb_ in low shear conditions ($\dot{\gamma }$ < 45 s^-1^) reflected the influence of RBC aggregation. It has been reported that arterial blood viscosity is lower than venous blood viscosity [34]. Since whole blood samples were withdrawn from the femoral artery for viscosity measurement in the present study, the apparent blood viscosity in the venular network could be higher than the viscosity shown in Fig 6.

The present study showed that a substantial reduction in the relative venular flow was discernible by decreasing MAP down to 30 mmHg (Fig 5B). This systemic pressure-dependent reduction in the venular flow with dextran infusion implied that RBC aggregation could contribute largely to the velocity reduction at such low flow conditions. As seen in the Fig 6B, RBC aggregation seemed to be the dominant factor in elevating flow resistance in the venular network [5]. In a comprehensive review on the hemodynamic effects of RBC aggregation by Baskurt and Meiselman [35], they stated that the effect of RBC aggregation on venous flow resistance could vary with different levels of RBC aggregation. A previous study by Yalcin *et al*. [36] reported a triphasic response of flow resistance to the level of RBC aggregation in the isolated hind limb of guinea pigs. In their study, a significant increase in flow resistance was observed at a moderate aggregation level of 97% increase in erythrocyte sedimentation rate (ESR), followed by a reduction at enhanced ESR of 136% and 162%. Subsequently, increasing ESR by 200% resulted in an increase in flow resistance again. The plasma-dextran concentration achieved in this study (~0.78%) was similar to a pathological human level [4], and venular flows were substantially decreased by reducing the MAP down to 30 mmHg. While the augmented cell-free layer formation by RBC aggregation could lead to a reduction in the resistance by lowering the effective viscosity, this could be negated by the sedimentation of RBC aggregates and the disruption of cell-free layer formation due to frequent branching in the venular network [35].

### Potential limitations

In the present study, a constant exposure time (10 ms) was used for all MAPs. This could decrease the sensitivity of our LSCI-based FVD measurement as compared to previous studies [22] which had used a longer exposure time (1000 ms). However, utilizing such a long exposure time was infeasible with our low-cost system as the pixel intensity of the raw speckle image was saturated at 1000 ms. In addition, fast flow generally requires a shorter exposure time for better spatial resolution and vice versa. Therefore, the spatial resolution of LSCI could be improved by adopting the multi-exposure LSCI [37,38], in particular under low flow conditions. In spite of the potential limitations, the flow measurement of our LSCI system, based on the conservation of volume flow at bifurcations, demonstrated a relatively high accuracy with errors of 4.0 ± 2.8% regardless of MAP.

## Conclusion

In summary, using LSCI, we successfully visualized and quantified the blood perfusion in venules at a network level. Our results highlighted that dextran infusion could reduce the FVD, in particular at low MAPs (30–70 mmHg). Moreover, the reduction in venular flow velocity by decreasing MAP was further impaired by dextran infusion, in particular under low shear conditions (MAP ≤ 40 mmHg). Blood viscosity analyses demonstrated that the further impairment in the venular network perfusion could be dominantly attributed to the formation of RBC aggregates in low shear conditions whereas the dextran-induced elevation of *μ*_pl_ could partly contribute to the impairment in high shear conditions.

## Supporting Information

S1 FigSpeckle contrast (*K*) as a function of *T*/*τ*_*c*_.
*T*/*τ*_c_ represented by the asymptote (simplified algorithm, Eq 3), Lorentzian (Eq 4), Gaussian (Eq 5), and alternative Gaussian (Eq 6) velocity distribution assumptions. The asymptote provides an identical contrast value when *K* is smaller than 0.6 (**Figure A**). Probability distributions of *K* over the entire set of speckle contrast images used in the present study. All the analyzed speckle contrast images satisfied the criterion for the use of asymptote (*K* < 0.6) (**Figure B**).
$$K^{2}=\frac{\tau_{C}}{2T}$$(3)

$$K^{2}=\frac{\tau_{C}}{2T}\left\{1-\text{exp}\left(\frac{-2T}{\tau_{C}}\right)\right\}$$(4)

$$K^{2}=\frac{\pi }{2}\frac{\tau_{C}}{T}\text{erf}\left(\frac{T}{\tau_{C}}\right)$$(5)

$$K^{2}=\frac{\tau_{C}}{2T}\text{erf}\left(\frac{\sqrt{\pi }T}{\tau_{C}}\right)$$(6)
(TIFF)

S2 FigFlow phantom study for obtaining a convergent factor between mean 1/*τ*_*c*_ and mean velocity.Cross-sectional view of the microchannel (**Figure A)**. Typical example of 1/*τ*_c_ map obtained by LSCI with a mean flow velocity of 8.23 mm/s in the microchannel (**Figure B**). Mean 1/*τ*_*c*_ as a function of mean velocity (**Figure C**). The mean velocity was calculated based on the relation ($\bar{v}\;=\;Q/A$) between a given volumetric flow rate (*Q*) and cross-sectional area of the channel (*A*). The solid line indicates the curve fitting of the experimental data (*y* = 9.62*x*, *R* ^2^ = 0.94, *n* = 4). Scale bar = 500 μm.(TIFF)

S3 Fig Determination of functional vascular density (FVD). Functional vascular density was defined as the total length of all vessel segments over the area of image of microvasculature. The speckle contrast image (**Figure A**) was converted into binary image (**Figure B**) and subsequently skeletonized (**Figure C**) to obtain the FVD. Scale bar = 500 μm.
(TIFF)

S4 Fig Venular diameter before and after dextran infusion at normal and reduced MAPs. There was no significant difference in venular diameter (ID = 19.3–40.5 μm) before and after dextran infusion regardless of MAPs (n = 9).
(TIFF)

S1 Text Validation study. (DOCX)
