# Supplementary figures and images for: Alteration of Blood Flow in a Venular Network by Infusion of Dextran 500: Evaluation with a Laser Speckle Contrast Imaging System
Source: PLoS One. 2015 Oct 14;10(10):e0140038. doi: 10.1371/journal.pone.0140038 (PMC4605724; doi:10.1371/journal.pone.0140038)

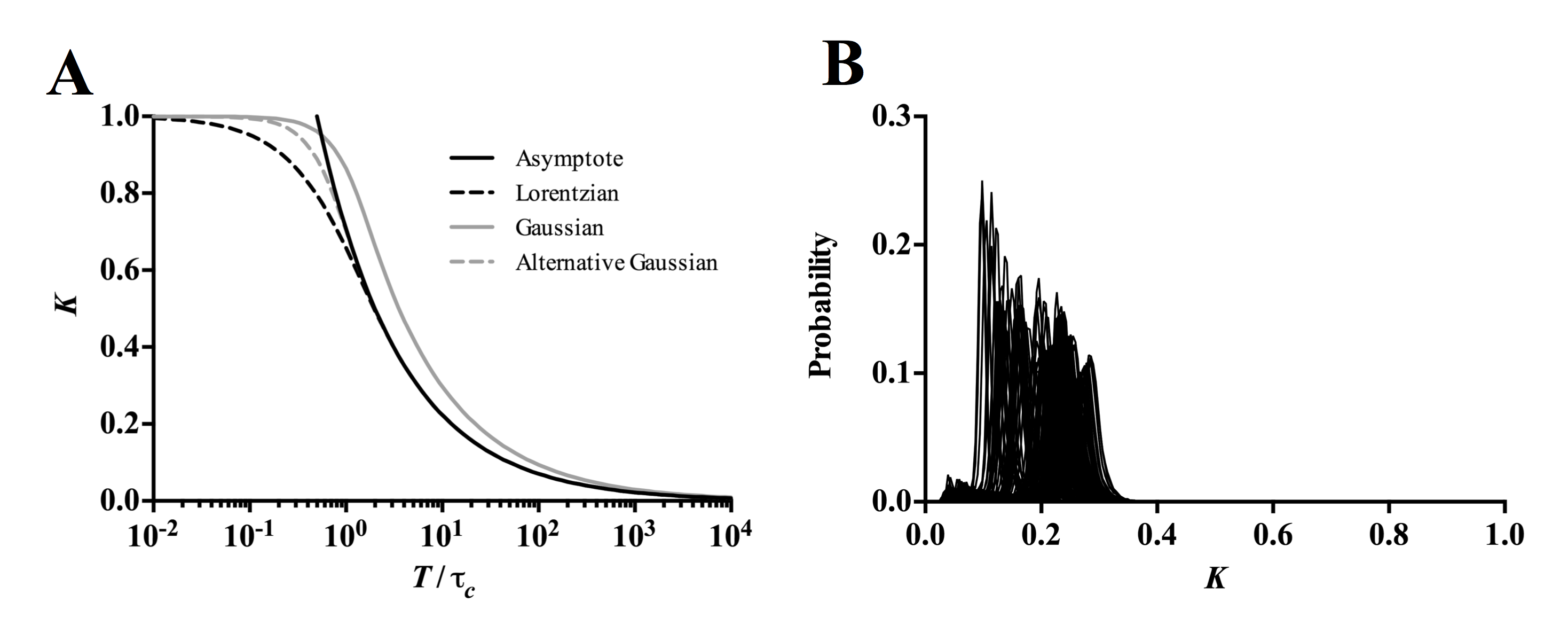

Supplement: S1 Fig — T/τ c represented by the asymptote (simplified algorithm, Eq 3), Lorentzian (Eq 4), Gaussian (Eq 5), and alternative Gaussian (Eq 6) velocity distribution assumptions. The asymptote provides an identical contrast value when K is smaller than 0.6 (Figure A). Probability distributions of K over the entire set of speckle contrast images used in the present study. All the analyzed speckle contrast images satisfied the criterion for the use of asymptote (K < 0.6) (Figure B). K2=τC2T(3) K2=τC2T{1−exp(−2TτC)}(4) K2=π2τCTerf(TτC)(5) K2=τC2Terf(πTτC)(6) (TIFF) [file pone.0140038.s001.tiff]

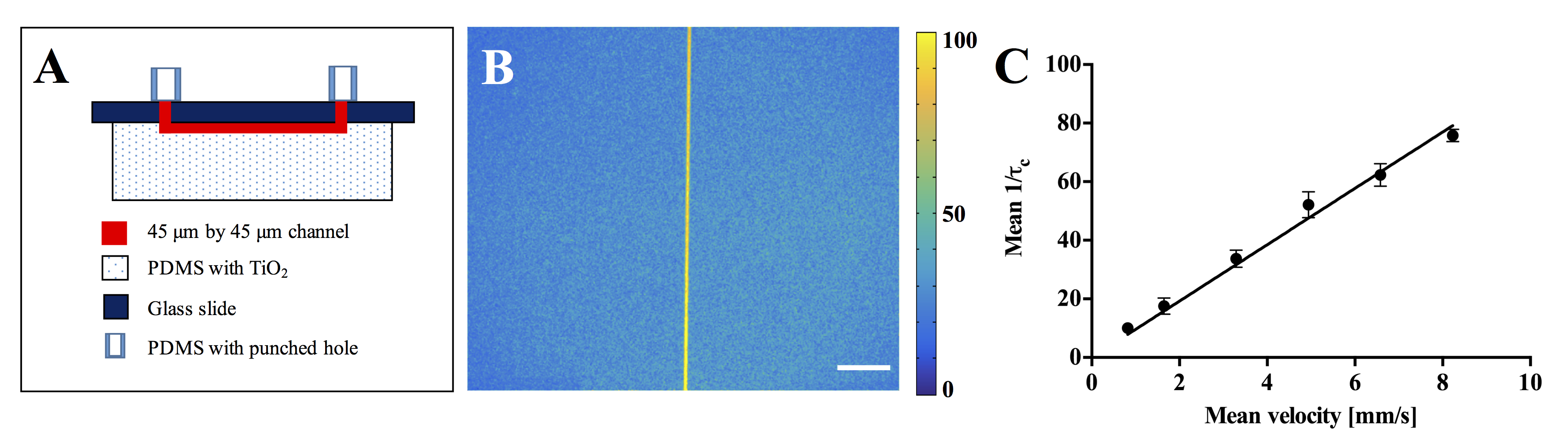

Supplement: S2 Fig — Cross-sectional view of the microchannel (Figure A). Typical example of 1/τ c map obtained by LSCI with a mean flow velocity of 8.23 mm/s in the microchannel (Figure B). Mean 1/τ c as a function of mean velocity (Figure C). The mean velocity was calculated based on the relation (v¯=Q/A) between a given volumetric flow rate (Q) and cross-sectional area of the channel (A). The solid line indicates the curve fitting of the experimental data (y = 9.62x, R 2 = 0.94, n = 4). Scale bar = 500 μm. (TIFF) [file pone.0140038.s002.tiff]

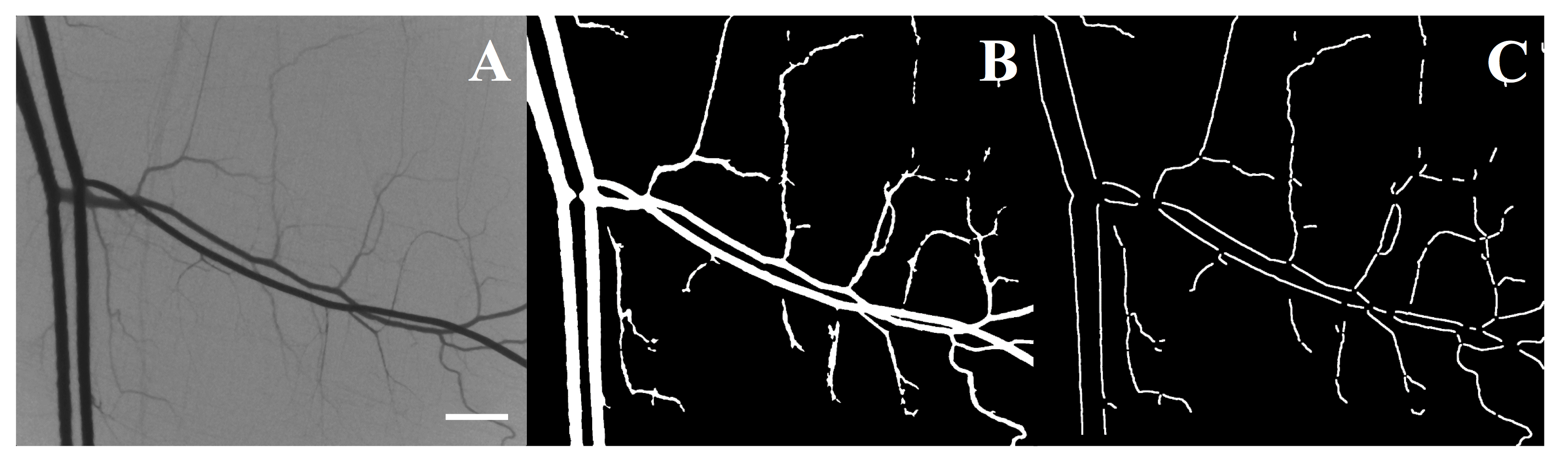

Supplement: S3 Fig — Functional vascular density was defined as the total length of all vessel segments over the area of image of microvasculature. The speckle contrast image (Figure A) was converted into binary image (Figure B) and subsequently skeletonized (Figure C) to obtain the FVD. Scale bar = 500 μm. (TIFF) [file pone.0140038.s003.tiff]

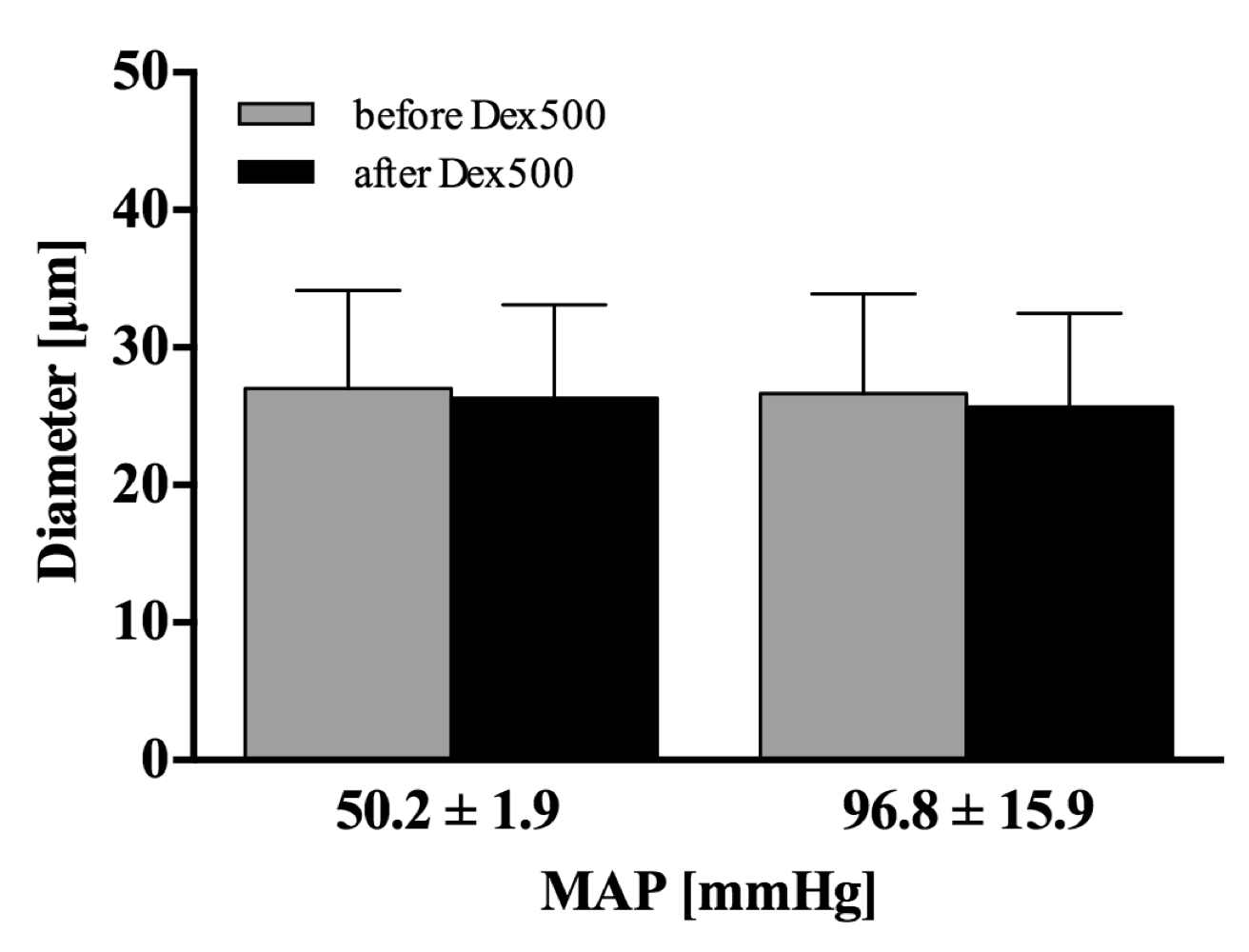

Supplement: S4 Fig — There was no significant difference in venular diameter (ID = 19.3–40.5 μm) before and after dextran infusion regardless of MAPs (n = 9). (TIFF) [file pone.0140038.s004.tiff]
