# Supplementary material for: Alteration of Blood Flow in a Venular Network by Infusion of Dextran 500: Evaluation with a Laser Speckle Contrast Imaging System
Source: PLoS One. 2015 Oct 14;10(10):e0140038. doi: 10.1371/journal.pone.0140038 (PMC4605724; doi:10.1371/journal.pone.0140038)
Supplement: S1 Text — (DOCX) [file pone.0140038.s005.docx]

**S1 Text. Validation study**

A microfluidic channel was used as a flow phantom to obtain the convergent factor (*α*) between correlation time (*τ*_c_) and absolute mean velocity (*v*). A polydimethylsiloxane (PDMS) microfluidic channel with a rectangular cross-section of 45 μm by 45 μm (width and height) was fabricated by the standard soft-lithography technique. To mimic static light scattering in tissue, titanium dioxide (TiO_2_, Sigma-Aldrich, St Louis, MO) was dispersed into 184 Sylgard PDMS gel (Dow Corning, Midland, MI) at 1 mg/ml [1]. As the application of LSCI was confined to the analysis of superficial flow, the phantom was placed upside down on the microscopic stage in order to obtain the speckle contrast images of the flow. In doing so, sample sedimentation could manifest especially in a conventional tubing-PDMS connection. To eliminate the possible effect of RBC sedimentation during the phantom study, two holes were drilled on the glass slide for the inlet and outlet reservoirs. A small piece of PDMS with a punched hole was then aligned and plasma bonded onto each glass hole to connect tubes (Figure A in S2 Fig). Rat RBCs at 35% hematocrit in PBS 1X was perfused in the channel at different flow rates (*Q* = 0.1 − 1 µl/min) using a syringe pump (KD Scientific Inc., MA). Subsequently, the mean 1/*τ_c_* was obtained by averaging the values from 10 analysis lines across the width of the microchannel which were evenly spaced along its entire length. Finally, the function between the mean 1/*τ_c_* and the mean velocity (, *A*: cross-sectional area of the channel) was then obtained from the phantom study (Figure C in S2 Fig) to establish the convergent factor (*α* = 9.62).

[1] Meisner JK, Sumer S, Murrell KP, Higgins TJ, Price RJ. Laser speckle flowmetry method for measuring spatial and temporal hemodynamic alterations throughout large microvascular networks. Microcirculation 2012; 19: 619-631.
